# Improving Chronic Disease Self-Management by Older Home Health Patients through Community Health Coaching

**DOI:** 10.3390/ijerph15040660

**Published:** 2018-04-02

**Authors:** Cheryl Dye, Deborah Willoughby, Begum Aybar-Damali, Carmelita Grady, Rebecca Oran, Alana Knudson

**Affiliations:** 1 Institute for Engaged Aging, 2037 Barre Hall, Clemson University, Clemson, SC 29634, USA; 2 School of Nursing, 417 Edwards Hall, Clemson University, Clemson, SC 29634, USA; willoud@clemson.edu; 3 Department of Recreation, Tourism and Therapeutic Recreation, Memorial Hall 119, Winona State University, Winona, MN 55987, USA; baybardamali@winona.edu; 4 Walsh Center for Rural Health Analysis, NORC at the University of Chicago, 4350 East-West Hwy, Bethesda, MD 20814, USA; carmelitagrady@yahoo.com (C.G.); oran-rebecca@norc.org (R.O.); knudson-alana@norc.org (A.K.)

**Keywords:** aging, chronic disease management, care transition, health coaching

## Abstract

The purpose of the study was to pilot test a model to reduce hospital readmissions and emergency department use of rural, older adults with chronic diseases discharged from home health services (HHS) through the use of volunteers. The study’s priority population consistently experiences poorer health outcomes than their urban counterparts due in part to lower socioeconomic status, reduced access to health services, and incidence of chronic diseases. When they are hospitalized for complications due to poorly managed chronic diseases, they are frequently readmitted for the same conditions. This pilot study examines the use of volunteer community members who were trained as Health Coaches to mentor discharged HHS patients in following the self-care plan developed by their HHS RN; improving chronic disease self-management behaviors; reducing risk of falls, pneumonia, and flu; and accessing community resources. Program participants increased their ability to monitor and track their chronic health conditions, make positive lifestyle changes, and reduce incidents of falls, pneumonia and flu. Although differences in the ED and hospital admission rates after discharge from HHS between the treatment and comparison group (matched for gender, age, and chronic condition) were not statistically significant, the treatment group’s rate was less than the comparison group thus suggesting a promising impact of the HC program (90 day: 263 comparison vs. 129 treatment; *p* = 0.65; 180 day 666.67 vs. 290.32; *p* = 0.19). The community health coach model offers a potential approach for improving the ability of discharged older home health patients to manage chronic conditions and ultimately reduce emergent care.

## 1. Introduction

In 2014, one in every seven Americans—46.2 million United States residents—was 65 years or older. By 2060, the number will increase to more than twice the number in 2014 [1]. As the United States experiences an increase in the proportion of adults over 65, the country will also see an increase in the number of individuals burdened with chronic conditions as 45.4 percent of Americans ages 65 or older reported two or three chronic health conditions and 14.1 percent reported four or more chronic health conditions in 2012 [2]. Effective self-management of chronic conditions is key to healthy aging [3].

This chronic disease burden will not be equally distributed throughout the United States. Approximately 46 million Americans, or 19.3%, live in rural areas of the nation [1]. Rural Americans, when compared to their urban counterparts, have consistently poorer health outcomes, a higher prevalence of illness, and are more likely to prematurely die from the five leading causes of death [4,5]. These health outcomes are consistently poorer due in part to a higher prevalence of risk factors and to lower socioeconomic status, insurance coverage, and access to quality health care [6,7,8,9,10]. Accessing care is especially difficult for older rural residents as their higher prevalence of illness requires more frequent healthcare and they must travel longer distances to receive it [11,12,13,14]. 

### 1.1. Chronic Disease Management

As reported earlier, in 2012 almost 60% of those over age 65 had two or more chronic health conditions, (i.e., hypertension, cardiovascular disease (CVD), congestive heart failure (CHF), stroke, diabetes (DM), cancer, arthritis, hepatitis, weak or failing kidneys, current asthma, or chronic obstructive pulmonary disease [COPD]) [2]. Chronic diseases can lead to losses of quality of life and productivity and, if they are not effectively managed, can result in acute and long-term complications requiring expensive hospitalizations and readmissions [15,16,17,18,19]. Nearly one in five Medicare patients discharged from the hospital is readmitted within 30 days, at a cost of over $26 billion every year [20]. The need to reduce readmissions has received heightened attention due to Medicare’s Hospital Readmission Reduction Program which penalizes hospitals with relatively higher rates of Medicare readmissions for diagnoses such as heart attack, heart failure, and pneumonia [21]. Fortunately, older adults can learn to successfully manage the chronic diseases related to many of these readmissions if they understand their disease process and master self-management tasks such as making lifestyle changes and adhering to medication regimens [22,23,24,25,26,27,28].

Home health services (HHS) after hospitalization can better prepare older adults and their caretakers to manage chronic diseases at home. However, HHS is primarily focused on delivery of skilled nursing care and during an episode of care it is challenging to find adequate time to mentor patients and caregivers in mastering disease self-management skills. Research has shown that inadequate control of chronic disease is often not due to a lack of technology or access to specialists, but rather due to a lack of support for daily lifestyle changes such as keeping records of glucose levels or increasing physical activity [23,24].

### 1.2. Health Coaching

Healthcare systems are now more accountable for patient outcomes because of shifts to value-based programs [29], which makes reduction of preventable ED use and hospital readmissions even more critical. Consequently, different roles have emerged to complement delivery of healthcare services and improve outcomes of the highest-risk patients. For example, patient navigators assist patients by eliminating barriers to timely diagnosis and treatment [30] and care transition managers and nurse health coaches facilitate post-discharge planning and care coordination to reduce hospital readmissions [31]. In the corporate sector, employee wellness programs are offered to help reduce healthcare costs [32]. In the community, Community Health Workers (CHWs), have historically built on their cultural competence to advocate for improved health outcomes of underserved community members [33]. Health coaching is a health education method focused on lifestyle changes delivered in a coaching context which has proven to improve chronic condition self-management [34]. Health coaches have been used in various settings such as healthcare systems, community, and corporate [34].

### 1.3. Health Coaches for Care Transition

A consortium comprised of a hospital, university, health department, and community-based organizations collaborated in planning the Health Coaches for Care Transition HCCT) program focused on improving the transition of older patients with chronic diseases discharged from Home Health Services (HHS) to home in order to reduce avoidable hospital readmissions and emergency department (ED) use. The program was funded by Health Resources and Services Administration (HRSA) and pilot-tested in Oconee County, South Carolina from 2006–2010. The consortium hospital is the only one in the county and it provided three years of data (2001, 2002, 2003) used in the HRSA grant proposal. According to that data [35], county residents over the age of 60 years were targeted for participation in the program because they had higher rates of many chronic diseases than their state and national counterparts, higher rates of emergent and hospital care, and the percentage of discharged HHS patients over the age of 60 years admitted to the ED was greater than the average of all South Carolina counties. Hospital ED admission data revealed that for the three-year period preceding the Health Coach program, the top three causes for admission were CVD—range 32.09% to 36.90%; CHF—range 40.73% to 47.16%; and DM—range 38.60% to 41.34% [35].Therefore, the consortium decided an intervention to reduce hospital readmissions should prioritize discharged HHS patients over age 60 who had a diagnosis of CVD, CHF, and DM with a program to improve self-management of those diseases. The program was also designed to reduce admissions for pneumonia, flu and falls.

Before the pilot project was implemented, the research team conducted focus groups with older community members to inform program planning [36]. Focus group participants indicated they needed help with chronic disease self-management and that a trained person from the community would be appropriate. The participants also preferred that this person should be called a Health Coach (HC). 

Components of the Chronic Care Model (CCM), which address improvement of healthcare at the community, organization, practice and patient levels [37], were incorporated into major aspects of the project. To support this collaborative approach, HHS Registered Nurses (RNs) were trained in the CCM and in ways the HC could assist their discharged patients in meeting individualized self-management goals established by the HHS RN and patient. 

Research questions addressed in the pilot study using a quasi-experimental design included:

1. Will discharged HHS patients over the age of 60 years with cardiovascular disease (CVD), congestive heart failure (CHF), or diabetes (DM) who are paired with a trained community-based HC be able to demonstrate self-management skills such as tracking their health conditions in a Health Diary?
2. Will discharged HHS patients paired with a HC have fewer admissions to the hospital and ED for the same diagnostic category as the original hospitalization compared to a group of discharged HHS patients matched for age, gender, diagnoses and period of HHS services?
3. Will discharged HHS patients paired with a HC have fewer hospital and ED admissions related to falls compared to a matched comparison group?
4. Will discharged HHS patients paired with a HC have fewer hospital and ED admissions related to pneumonia compared to a matched comparison group?
5. Will discharged HHS patients paired with a HC have fewer hospital and ED admissions related to flu compared to a matched comparison group?
6. Will average costs of hospital or ED admissions for HC clients be less than the average cost of admissions of those in the comparison group?

## 2. Materials and Methods

All aspects of the Health Coaches for Care Transition program, including informed consent documents obtained from the volunteer HCs and discharged HHS patients included in the study, were approved by the Clemson University Internal Review Board. A quasi-experimental design was used to evaluate the effectiveness of the pilot project. Process measures included: number of community members trained to be HCs; number of HCs who continued throughout the project period; number of referrals to the program from HHS RNs; number of referrals who agreed to participate in the program; and reasons why referred patients did not participate in the program. Impact measures included select OASIS (Outcome Assessment and Information Set-B1) measures used by HHS which were also administered by the HCs. These indicators included: level of physical activity; adherence to special or therapeutic diet; usual food intake pattern; grooming; ability to dress upper body; ability to dress lower body; bathing; toileting; transferring; ambulation/locomotion; feeding or eating; planning or preparing light meals; transportation; laundry; housekeeping; shopping; ability to use telephone; medication compliance/education; tobacco use; immunization received within the past 12 months; home modifications including structural barriers, safety hazards, and sanitation hazards; and ability to engage in self-management behaviors such as tracking health condition in Health Diary for at least two weeks. Outcome measures included; readmission to the hospital or ED for original diagnostic category of CVD, DM, or CHF; admission to hospital or ED for fall, flu, or pneumonia; and costs of admissions. 

### 2.1. Health Coach Recruitment

Community members were recruited to be trained as a HC by the project Community Coordinator who used several outreach strategies such as presentations at civic organization meetings and advertising in church newsletters and local newspapers. They were recruited from rural communities with large percentages of older adults so that they might better understand local norms and barriers related to accessing healthcare and managing chronic conditions experienced by the priority population. The volunteers eventually selected for training passed criminal background checks and were evaluated through structured interviewing techniques recommended for social services volunteer selection [38]. 

### 2.2. Health Coach Training

A training curriculum was developed to include chronic disease content, select CHW competencies, and health educator skills. It also incorporated communication strategies and adult learning principles, within an overall philosophy of self-determination where the patient defines their own vision of optimal health and works collaboratively with their health coach in a mutually respectful relationship to develop action plans for achieving their goals [39]. As part of their training, HCs were certified by Clemson University research compliance staff in human subject protection since HCs obtained informed consent and collected data from study participants.

Skill development in CHW competencies such as accessing community resources and assisting in making healthcare appointments [33], were included in the training since CHWs have proven to be a cost-effective strategy in facilitating individual’s adherence to recommended health behavior changes, self-management of health conditions, and access to health care [25,26].Part of the success of CHWs is their cultural competence acquired through membership in the communities they serve [33] which was considered in HC recruitment efforts.

HCs were trained in self-management best practices for cardiovascular diseases (CVD), hypertension, congestive heart failure (CHF), diabetes Type II (DM) and stroke. HCs were trained to teach and encourage their clients to monitor and record weight, blood pressure, pulse rate, and blood glucose, as indicated by the clients’ diagnoses; and to calibrate their relevant devices, all provided by the program, such as easy-to-read digital scale, BP monitor, and glucose monitor. HCs learned how to help clients maintain a log of their health status measures in their Personal Health Diary and how to use a “stop light” visual aid to recognize “red flag” symptoms such as shortness of breath, cough, and swelling of the feet by those with CHF, and how to conduct foot self-examinations to detect lesions by those with DM. By learning to recognize red flags of disease progression, clients were alerted to arrange early healthcare intervention to prevent an ED visit and/or hospitalization. 

HCs were also trained in health education competencies so they could effectively mentor their clients to make lifestyle behavior changes. They developed skills in helping their clients build self-efficacy, develop behavior change goals, reward themselves for meeting goals, and prevent relapse to unhealthy behaviors. These skills helped HC clients improve their success in mastering self-management behaviors such as following dietary recommendations, creating a physical activity plan appropriate for their health status and fitness level, and selecting tobacco use cessation strategies. Additionally, HCs learned how to assist someone in conducting a home fall risk and safety assessment and how to secure community resources to make home repairs. 

Consistent with the Chronic Care Model [37], HCs were also trained to activate and prepare their clients to have more interactive medical visits with their primary care providers (PCP) through actions such as reporting disease symptoms; inquiring about medication use and side effects and reporting side effects; and bringing a list of concerns and questions for discussion during PCP visits. HC clients also regularly brought their Personal Health Diary to PCP visits, a practice which was well received by the PCP as it provided day-to-day data about their patient’s health and demonstrated that their patient was actively engaged in their own health management.

Finalized training curriculum modules included: (1) Parameter and Role of Health Coach; (2) Safety and Fall Prevention; (3) Adult Learning Principles and Communication Skills; (4) Psychosocial and Physical Aspects of Aging; (5) Cardiovascular System; (6) Heart Disease and Stroke; (7) Diabetes; (8) Pneumonia and Flu; (9) Medications and Self-management; (10) Chronic Disease Self-Management Behaviors (Nutrition, Physical Activity, Stress Management and Tobacco Use Cessation); (11) Changing and Maintaining Health Behaviors; (12) Identifying and Accessing Community Resources such as smoking cessation classes, free immunizations, utility bill waivers, and home modification assistance; and (13) Human Subjects Protection. 

To pass the 30-h training and become certified, HC candidates were required to attend all sessions and to achieve a score of 80% or greater on the knowledge test. The Project Director and Community Coordinator conducted six separate training sessions over the four-year project period including 51 people with 43 meeting requirements to be certified as Health Coaches and accepting clients. Knowledge test scores for all 51 trainees averaged 61.2% at pretest and 80.0% at post-test. Of the 43 certified HCs, 38 were females and five were males with 25 Health Coaches (59.5%) continuing to serve clients through all four years of the project period. Some HCs preferred to work as teams and others worked alone.

### 2.3. Health Coach and Patient Assignment

Certified HCs who agreed to take clients were paired with discharged HHS patients. The HHS RNs determined which of their patients met program criteria of age and chronic condition as well as who lacked adequate assistance as determined by the Outcome and Assessment Information Set (OASIS) used in HHS. They informed qualified patients about the HC program and, if the patient was interested, obtained their signature on a Health Information release form which granted permission to release their self-care plan to a HC. The RN then faxed their own phone number and the patient’s name, home address and discharge date to a dedicated fax machine in the Project Directors locked office. The Director then contacted a HC who lived near the HHS patient to see if they were available to work with a client over the next four months. If the HC agreed to take a client, the Director asked that they call the HHS RN to arrange a meeting between the HHS RN and patient at the patient’s residence during the final HHS visit before discharge. The Director then provided the assigned HCs name to the HHS RN who shared it with their patient. At the joint HHS visit, the HHS RN introduced their patient to the HC and explained that HCs were trained to help patients follow-through on the self-care plan developed during HHS. This personal introduction of the HC by the HHS RN is an adaptation of the “warm hand-off” strategy important to successful patient care transition from one provider to another [40]. HHS RNs included the name of their patient’s HC in his/her discharge report to the referring physician. This identification was important in situations when a HC contacted the physician’s office on behalf of their client such as when the client’s emergency care plan was implemented. 

### 2.4. Health Coach Activities

On the first visit to the patient’s home after HHS discharge, the HC provided an overview of the HC program including a clear explanation of the limitation of their role and the time they would spend with the client which did not exceed four months in most cases. If the patient had a caregiver, this person was also invited to attend the initial session and all those which followed. The HC explained that the goal was for the client to be able to follow their self-care plan developed during HHS. If the client was still interested in the program, the HC then read through an informed consent document, provided two copies of the form to the patient, and asked him/her to sign one copy which was sent in to the Project Director. The HC transferred relevant information from the patient’s HHS self-care plan such as their parameters for acceptable glucose levels to a simpler Personal Health Diary that was then used by the patient to log their health status. At the first or second home visit, the Health Coach also collected selected Outcome and Assessment Information Set (OASIS) data such as edema, drainage, shortness of breath, cough, chest pain, dizziness, cramping, pain or temperature change in lower extremities, dietary behavior, physical activity, smoking levels, ability to engage in ADLs and IADLs, medication compliance behaviors, immunizations and residential hazards or structural barriers which might increase risk for falls. 

The HC met with their assigned discharged HHS patient for a total of approximately 3.5 h per week in month 1 (two home visits and three phone calls); 3 h per week in month 2 (one home visit and four phone calls); 2.5 h per week in month 3 (no home visits with four phone calls; and 2 h per week in month 4 (four phone calls). The number of contact hours were tapered from the beginning to the end of HC services in order to promote independence from the HC. If by the end of the 4th month of HC support, self-care did not seem probable, with client permission, the HC placed the client’s name on the Community Long Term Care waiting list or contacted the HHS RN about the possibility of recertification for HHS. Six months after the end of Health Coaching (month 10), the Health Coach made one final home visit to collect OASIS data.

During the first home visit, the HC provided their client with a notebook of materials relevant to their chronic disease including a “stoplight” of disease symptoms and recommended actions. They also provided a digital blood pressure cuff, digital scales or digital glucose monitor, according to client needs, and instructed them in how to use the equipment. When the client demonstrated he/she could properly use their equipment, they recorded their baseline health status indicators such as BP, glucose levels or weight in their Personal Health Diary. On subsequent home visits, the Health Coach reviewed with the client their daily entries in the Personal Health Diary. Twenty-one out of the 33 Health Coach clients regularly tracked their conditions in their Health Dairies for a range of days from 21 to 224. With those clients not regularly tracking their condition, Health Coaches discussed ways to add this task to their daily activities. For those clients who were successfully tracking, the Health Coach complemented them on their commitment to monitoring their health, celebrated successes and discussed any trends which may be problematic. If, for example, the client was not staying within their recommended glucose parameters, the HC discussed any difficulties the client may be having with diet and physical activity or with taking diabetes medications. In this same example, if glucose levels were above those recorded on the “stoplight” of red flag symptoms, the HC helped the client follow through with the recommended action.

During the four months of home visits and phone calls, the HC tailored activities to the needs of the client and/or their caregivers. These activities included: improving chronic disease self-management skills; coordinating health care services and provider referrals; collaborating with community organizations to obtain resources such free immunizations, utility bill waivers, food stamp applications, home repairs, and meal deliveries; helping clients develop a medication management plan; arranging and reminding clients about appointment schedules and treatment regimens; making transportation arrangements for medical appointments and pharmacy visits, food shopping or physical activity programs; facilitating communication between client, family, caregivers, and service providers; and acting as an advocate with the PCP or HHS RN.

In addition to contacts with their clients, HCs attended monthly meetings as a group and made weekly contact via phone or e-mail with the Project Director during the time they were serving clients. At the monthly meetings, HCs submitted a client contact log and travel log if they were requesting reimbursement of travel expenses. They reported on their client’s progress in reaching self-management goals and their ability to collect and record their health information in their Personal Health Diary. They also shared challenges and solutions related to working with the clients and their families. The HCs, most of whom were retired, anonymously reported through an open-ended question survey administered in year 3 and 4 of the project that they experienced positive impacts from the program such as role satisfaction and fulfillment of their desire to help others in a meaningful way (quotations from HCs are included in Results section). The HCs also enjoyed getting acquainted and making new friends with their peer HCs.

The HCs submitted data collected with the OASIS and the client’s Personal Health Diary to the Project Director. The Project Director assigned a random number to each HC client which was linked with the client’s OASIS and Personal Health Diary information. A data analyst then entered, managed and analyzed the data. The Project Director worked with the HHS Director to match HHS patients who chose not to participate in the HC program on gender, age, health condition, and period of HHS services with those HHS patients who received HC assistance. Those HHS patients who served as the comparison group were also assigned random numbers. The HHS Director then provided information about hospital readmission, ED use and related costs of both the treatment and comparison group members to the Project Director. Data were then analyzed in the aggregate using assigned random numbers to determine differences in readmissions, ED use and costs between the treatment and comparison groups. See Table 1 for activities of HHS RN, HCs, and Project Director.

### 2.5. Data Analysis

The researchers used SPSS [41] for data analysis. This study used a quasi-experimental design with a treatment and comparison group (non-equivalent control group) with comparisons of ED/hospital visits after HHS discharge of study participants to 90 days and 180 days. Since a person’s chronic diseases and number of diseases is indicative of their overall health as well as their likelihood of ED/hospital visits and resultant costs, a careful review of the HHS diagnoses of the treatment group participants and the comparison group members was conducted and it revealed an imbalance in types and number of conditions. Therefore, weighting was used by the type and number of conditions to balance the two groups. 

### 2.6. Sample

After HCs were certified and referral protocols were in place, HHS RNs began referring eligible patients to the program. Patients enrolled in HHS between 22 August 2006 and 20 August 2008 were referred and enrolled in the HC program between 30 November 2006 and 26 September 2008 after HHS discharge (See Table 2 and Table 3). The HHS Director matched HHS patients who were eligible for the HC program but chose not to participate on gender, age, health condition, and period of HHS services with HC program participants. All program participants (aged 61 to 96 years) and comparison group members (aged 62 to 91 years) had a diagnosis of congestive heart failure (CHF), diabetes Type II (DM) or cardiovascular diseases (CVD) such as hypertension (see Table 2, Table 3, Table 4, Table 5 and Table 6). There were 65 referrals to the program from HHS RNs, but 12 were not accepted because they did not reside in a rural census tract as required by the funder, they did not have a primary diagnosis of CVD, CHF, or DM, or they were not 60 years of age or older. Out of the 53 clients served in the HC program, complete data were collected at two months, four months, and 10 months post HHS discharge from 33 program participants. Reasons for incomplete data collection included: dropping out of program, moving out of the geographic region, admission to nursing home care, and cognitive impairment. See Table 2, Table 3, Table 4, Table 5 and Table 6 for HHS diagnoses and cause of emergent care of program participants and comparison patients.

## 3. Results

Of the 33 program participants with complete data, 14 (42.4%) received emergent care for at least one of their original HHS diagnoses of interest for an average cost of $11,161 (Table 3) as compared to 22 of the 36 members (61.1%) of the comparison group receiving emergent care for HHS diagnoses for an average cost of $21,523.68 (Table 5). Two additional comparison group members were admitted for emergent care but cost data were not available and the cases were not included in Table 5. It should also be noted that HC client #14 had an admission for a GI bleed costing $29,380 determined to be due to medication, but it is unknown whether the bleed was due to medication side effects or mismanagement. Regarding admissions for pneumonia, flu and falls during the project period (Table 6), the HC program participants had no admissions for falls or flu and only one of those clients with emergent care for their chronic condition was also admitted for pneumonia. In the comparison group, seven of those with emergent care for their chronic condition were also admitted for pneumonia, flu and falls with two additional admissions for falls only for a total of 24 out of 38 comparison patients receiving emergent care. The overall hospital and ED admissions for chronic conditions of CVD, DM, and CHF as well as for falls, pneumonia and flu were 14/33 (42.4%) for the HC clients and 24/38 (63.16%) for the comparison patients. See Table 2, Table 3, Table 4, Table 5 and Table 6.

Table 7 shows the average ED/hospital visit rates for 90 and 180 days for both the comparison and treatment groups as well as the average ED/hospital visit costs per patient for the comparison and treatment groups. For 90 days, the comparison group had an average ED/hospital visit rate of 0.28 whereas the treatment group had an average ED/hospital visit rate of 0.13. For 180 days, the average ED/hospital visit rate was 0.72 for the comparison group and 0.29 for the treatment group. For the total average ED/hospital visit costs per patient, for the comparison group, across 90 days, the total average cost of care was $1135.96 whereas the average cost of care for the treatment group over 90 days was $770.50. For 180 days, the total average ED/hospital visit cost per patient for the comparison group was $7203.68 and $2545.38 for the treatment group. The percent of comparison group patients who had an ED/hospital visit at 90 days was 24% while the treatment group percentage was 10%. Over 180 days, the percent of comparison group patients who had an ED/hospital visit was 44% while the treatment group percentage was 19%. Although differences in the ED/hospital visit rate between the treatment and comparison group were not statistically significant, the treatment group’s rate was less than the comparison group thus suggesting a promising impact of the HC program (90 day: 263 comparison vs. 129 treatment; p = 0.65; 180 day 666.67 vs. 290.32; p = 0.19).

Table 8 show the range of cost data used in the analysis.

HCs provided anonymous feedback about their experience in the third and fourth year of the project. Some HCs had to leave the program because of their own health challenges or those of their spouse, or because they moved. Of those HCs who continued, all feedback revealed that they were satisfied with their role and that they felt their training was adequate. An example of this feedback is below:

“I really enjoyed becoming a health coach, and will definitely maintain contact with my client even once our relationship ends. This program helps to close the gap between when a person leaves home health and must become independent in their care at home. It serves as another step to becoming self-confident and maintaining that independence. And, by linking lay people to persons in the community who could use that extra stepping stone, it truly does offer a benefit not only for the client, but for the health coach who gains countless rewards by serving others. I think it is so important to note that not only has the program helped the client, but your program helps those that you train to become health coaches. The self-awareness gained during the classroom sessions for those that are not fully familiar with chronic conditions/nutrition will help those community members as well. So, not only are you reaching “clients” but you are teaching those that are becoming health coaches to become more aware of their own lifestyles. Being a health coach is not easy by any means, you must realize that you are entering into someone’s personal home, you must not make judgements. You are to be an advocate. You must, at times, reteach the same material over and over, requiring much patience. But, when the client does make a change, no matter how small, you become their biggest fan, and that is AWESOME!”

Another Health Coach provided this feedback:

“I don’t think I would change anything about the experience itself. Coming into our particular client’s household, i do believe the client thought we were going to be giving them “medical care” and bringing them free things, taking them to all appt. and running errands whenever needed. I think it is so important to really define your role at this time. Also, you must communicate from the start that the relationship will end at a certain point. Define those boundaries on day 1! Other advice...be patient, repeat information often, be even more patient if the client does not change a particular habit and look into the reason for why the change was not made...make no judgements, be an advocate, listen openly, and have fun! and did I say, be PATIENT? Assisting the client with modification of lifelong bad habits will not happen overnight.”

## 4. Discussion

Representatives from the Atlanta Regional Division of the Office of Performance Review conducted a site visit of the program on 27 March 2008, as part of its effort to disseminate information about leading practices in the areas of clinical practice, outreach, cultural competence, administration, and other practices that are implemented within HRSA-funded programs. In their report, the representatives concluded that it was a promising model for reducing readmissions of elderly patients with chronic conditions and stated that, “What made this a leading practice is the training tool used by Oconee, their selection of dedicated trainees that are familiar with the local culture, and the continuity of care the program established by allowing the health coaches to partner with the nurses at the home health agency prior to the coaches taking over”. According to a report on U.S. Hospital Readmissions released in February 2013 by the Robert Wood Johnson Foundation, one in five elderly patients is back in the hospital within 30 days of leaving and many of these readmissions can be prevented [42]. The reasons discharged elderly patients are often readmitted is because they did not understand their illnesses or treatment plans and were unable to follow self-care instructions, including medication use and getting follow-up care. Family members are frequently not included in discharge planning, even though they may be central caregivers to the patient. Of the $26 billion annual cost of readmissions for Medicare patients, more than $17 billion is due to avoidable readmissions. Consequently, the Centers for Medicare & Medicaid Services has identified avoidable readmissions as one of the leading problems facing the U.S. health care system and now penalizes hospitals with high rates of readmissions for their heart failure, heart attack, and pneumonia patients [21]. 

The project described in this paper, Health Coaches for Care Transition (HCCT), is relevant to changes in Medicare reimbursement in that it proposes a new model for including a community-based program to enhance HHS outcomes and reduce hospital readmissions and ED use. Several interventions staffed by healthcare providers have shown positive effects on readmission rates [43,44]. Examples include discharge management with follow-up by an advanced practice nurse; patient coaching by a nurse; disease/health management programs provided by health educators; and telehealth services. HCCT demonstrates that a community-based program staffed by trained volunteers could be an important component of these efforts.

The HCCT program uses some of the same strategies developed by Dr. Eric Coleman as part of the Care Transitions Intervention (CTI) [44]. In both programs, participants are taught how to use a personal health diary/record, how to identify and appropriately respond to symptoms that their chronic condition may be worsening, how to develop a medication management system, and how to effectively communicate with healthcare providers. Differences in the programs include an average length of service of four weeks for the CTI and a length of four months for the HC program which allows more time for HCs to assist their clients in activities such as making lifestyle behavior changes, arranging for transportation, and accessing community resources such as utility waivers when clients struggled to pay heating/cooling bills, food stamps or home-delivered meals, and even adult protective services when elder abuse was suspected. The HCs found that education and support for the family caregiver(s) of their client was particularly important and they included them in their home visits, encouraged them to take a more proactive role in their loved one’s care, and provided them with information about how to access community resources to assist in their efforts. Another primary difference in the CTI and HC program is in staffing whereas trained community volunteers and a Community Coordinator are used in the HC program compared to the requirement of an advanced practice nurse in the CTI. It should be noted, however, that HCs were carefully trained regarding the parameters of their role which was to assist their clients in following the self-care plan developed by the HHS RN. 

The HCCT program also has similarities with a project developed by Russell et al. where home health aides were trained to serve as health coaches for home care patients with chronic illness [45]. The training curriculum was similar in content covering chronic conditions, medication management strategies, maintenance of a personal health record, and health behavior change strategies. In one project, trained home health aides worked with heart failure patients for an average of 30 days and in another they worked with patients with other chronic illnesses for an average of 84 days. In contract, HCCT coaches worked with clients for four months and most were not interested in career advancement as they were primarily volunteer retirees. Further, five of the 43 HCs were men as compared to home health aides who are primarily female. One of the biggest differences between the HCCT program and the heart failure program developed by Russell et al., is that the trained home health aides provided services alongside traditional post-acute skilled home care services for the first 30 days after hospitalization whereas in the HCCT program, HCs did not provide services until after patients were discharged from HHS because the HHS agency preferred that the services be delivered separately. This preference may be partly explained by the fact that the HCCT project was first delivered ten years ago when community-based models emphasizing integrated and holistic approaches to patient care were not as common in healthcare system practices and delivery of long-term care. Ten years ago, there was also less formalized support within healthcare systems for the inclusion of paraprofessionals such as Community Health Workers in healthcare teams. 

Using a community-based HC to reinforce the work of HHS RNs or advance practice nurses serving as a Care Transition Coach allows healthcare providers to target their efforts toward areas best managed by them, while the HCs assume the roles for which they have been trained. The HCCT program can be replicated in other rural areas because of its use of national models such as the CCM and CTI and use of materials which are available to the public. Participants in the HCCT program demonstrated their ability to monitor and track their chronic diseases; to make lifestyle changes; to reduce fall risk factors; and to access assistance from healthcare and community agencies. HC clients had fewer hospital and ED readmissions in the same chronic disease diagnostic category used in their original hospitalization and fewer admissions for flu, pneumonia and falls as compared to a group of HHS patients matched for gender, age, and chronic condition. Although differences in rehospitalization and ED use between the treatment and comparison group were not statistically significant, the results are clinically significant and suggest a promising impact of the HC program. 

The study reported in this paper has limitations. First, there may have been a self-selection bias as the HHS patients who agreed to participate in the program may have been more motivated to self-manage their condition than those who did not want to participate. In addition, the study only tracked ED use and readmissions to the same hospital providing the original patient care before the HHS episode of care. Health Coach clients verified with their HC that they had not used another hospital for emergent care during the project period, but it is possible that the comparison group members received emergent care from another hospital as well as the original hospital. Finally, the study had a small sample size which may have affected the results and the statistical significance of the results. Future studies should replicate this work with larger numbers of patients to better determine effectiveness. 

## 5. Conclusions

Previous research has examined the care transition challenges for older patients, particularly those with chronic disease, but few have looked at the use of community health coaches in mentoring discharged HHS patients to improve their ability to self-manage their chronic conditions [40,41,42]. This study demonstrates that trained, volunteer, community health coaches can be an effective tool in improving the transition of older, rural patients with chronic conditions from HHS to self-care at home. Although differences in the ED/hospital visit rate between the treatment and comparison group were not statistically significant, the treatment group’s rate was less than the comparison group thus suggesting a promising impact of the HC program (90 day: 263 comparison vs. 129 treatment; *p* = 0.65; 180 day 666.67 vs. 290.32; *p* = 0.19). Study data suggest that the Health Coaches for Care Transition program has potential to reduce hospital readmissions and avoidable ED use related to chronic condition management as 57.6% of program participants had no emergent care compared to 44.4% of comparison group members. Additionally, 1 of the 33 program participants with chronic conditions (3%) were admitted for pneumonia, flu or falls during the project period in contrast to 7 of the 36 comparison group members with chronic conditions (25%) admitted for these same incidents.

The program tested in this study is cost-effective as it can be staffed by trained volunteers and a Project Director/Community Coordinator with a yearly salary of about $35,000 with the provision of about $100 in materials and supplies per participant dependent upon the equipment needed to track their condition such as a blood pressure monitor. This program can be replicated in other communities as the Health Coach training curriculum, Health Coach manuals, and client notebooks are available through the lead author. Health Coaches for Care Transition demonstrates how a healthcare system, university, and community organizations can collaborate to promote the health of rural older adults through community-based health coaching for chronic disease self-management.

## Figures and Tables

**Table 1:** Activities of Project Staff.

Who	When	What
HHS RN	Before HHS discharge	Identify eligible patient and inform about program, obtain patient signature on health information release form, fax RN phone number and patient name, address and discharge date to Project Director
Project Director (PD)	After HHS referral	Contact HC in same geographic area as patient, if HC agrees to take client, PD asks HC to contact HHS RN to schedule home visit with RN and patient near the discharge date
Health Coach (HC)	After contact from PD	Call HHS RN to schedule patient meeting, meet HHS RN at patient home for last HHS visit
HHS RN	During last HHS visit	Introduce HC to patient, explain HC role in helping patient follow-through with care plan, include name of HC in patient discharge report to referring MD
HC	After HHS discharge	Schedule first visit with patient
HC	First client visit—week 1 after HHS discharge	Provide overview and goal of program, types of interactions (home visit and phone calls), length of time (approximately 4 months), obtain informed consent and return one copy to PD, transfer care plan information to Personal Health Diary and explain how to tract health status, discuss client personal goals, collect selected OASIS data on disease symptoms, provide “stoplight” with symptoms and recommended action, teach patient how to use equipment needed to track health indicators such as BP monitor, digital scales and glucometer and help them practice
HC	Client contact—weeks 2 to 12	Review Personal Health Diary, discuss any challenges in disease self-management, praise accomplishments, provide tailored activities according to client needs such as home repair or utility bill assistance, transportation, assist with scheduling medical appointments and developing medication management plan, communicate with MD or HHS RN
HC	monthly	Meet with other HCs and PD to discuss challenges and solutions, turn in client contact log of activities to PD
PD	ongoing	Assign random number to each client for use on Personal Health Dairy and OASIS data before giving to data manager
PD	Every six months	Meet with HHS Director to identify comparison group participants for data comparison with HC clients on hospital readmissions and ED use, and cost of care
Data analyst	Ongoing	Data entry, analysis

**Table 2:** Gender, Race, Age, HHS and HC Program Enrollment Dates, HHS Diagnoses of Health Coach Clients with No Emergent Care (*N* = 19/33, 57.6%).

Client#	Gender	Race	Age	Enrolled in HHS	Enrolled in HC Prog.	HHS Diagnoses
140			86	8/20/08	9/26/08	CHF, CVD
121	F	CA	79	2/14/07	8/13/07	left heart failure (CHF)
						benign hypertension (CVD)
137	M	CA	80	3/22/08	7/1/08	idio periph neuropat long term anticoag U cardiac dysrhythmia paralysis agitans hypertension NOS (CVD)
60	F	CA	77	1/25/07	8/13/07	chr pulmon heart dis long term anticoag U atrial fibrilliation (CVD)
61	F	CA	76	10/1/07	11/19/07	atrial fibrillation
						hypertension NOS (CVD)
						syncope & collapse
						reflux esophagitis
						DM2/NOS
139			66	8/7/08	8/26/08	hypertension NOS (CVD)
						DM2/NOS
19	F	CA	82	3/12/07	8/13/07	AB gait *
						decubitus ulcer butt
						muscle weakness
						urinary tract inf nod
						DM2/NOS W comp NOS N
991	F	CA	78	12/30/07	6/6/08	lower limb ulcer NOS
						AB gait *
						DM2/NOS W circ dis U
						long term anticoag U
						ther drug monitoring
17	F	CA	83	4/19/07	6/12/07	OCB W exacerbation
						DM2/NOS uncomp NSU
80	F	CA	63	8/5/07	9/27/07	OCB W exacerbation
						muscle weakness
						DM2/NOS uncomp NSU
15	M	CA	81	2/6/08	6/12/07	DM2 NOS uncomp UNC
						atrial fibrillation
						HX TIA/infarct W/O R
						late EFF CVD-dysphas
						hypertension NOS (CVD)
133	F	CA	84	3/11/08	7/14/08	DM2/NOS comp NOS U
						AB gait *
						hypoglycemia NOS
						atrial fibrilliation (CVD)
						long term anticoag U
136	M	CA	71	2/25/08	6/13/08	DM2/NOS uncomp NSU
						hypertension NOS (CVD)
						obesity NOS
						left heart failure (CHF)
						long term insulin US
35	F	CA	77	4/24/08	6/19/08	H zoster complicated
						OTH persist ment DIS
						DM2 NOS uncomp NSU
						hypertension NOS (CVD)
						CLL W/O remission
33	F	CA	66	5/10/07	7/23/07	statuspost
						muscle weakness
						DM2 NOS uncomp NSU
						benign hypertension (CVD)
						OCB W/O exacerbation
210			72	6/14/08	9/8/08	DM, CVD
81	M	CA	84	4/25/08	5/28/08	CHR SYS & diastolic
						OCB W exacerbation
						hypertension NOS (CVD)
						atrial fibrilliation
						hyperlipidemia NEC
51	M	CA	68	3/6/07	7/20/07	COR AS-graft type NO
						PERIPH vascular DIS (CVD)
						OCB W exacerbation
						recurrent MDD unspec
122	F	CA	80	7/25/07	7/20/07	adjust cardiac pacem
						altered mental status
						muscle weakness
						sinoatrial node DYSF (CVD)

* abnormality of gait.

**Table 3:** Gender, Race, Age, HHS Diagnoses, HHS and HC Enrollment Dates, Causes and Costs of Emergent Care of Health Coach Clients (*N* = 14/33, 42.4%).

Client#	Gender	Race	Age	Enrolled in HHS	Enrolled in HC Prog.	HHS Diagnoses	ED/Hospital Admission	Cost of Care
21	F	HS	91	8/22/06	11/30/06	senile degen brain,	10/28/07 end stage renal failure, htn, anemia	$9643
						HTN NOS (CVD)		
						anemia IN CKD		
						memory loss		
						CKD—stage 1		
135	F	CA	73	12/3/07	6/17/08	DM1 uncomp NSU	9/20/08 urinary problem	$897
						open wound of scapul, anemia NOS		
						long term insulin US, altered mental status		
13	F	CA	61	8/23/06	3/1/07	BK amputation status, DM2/NOS W neur manif, autonom neuropat IN, AB gait *	10/5/07 chf, acute renal failure	$6482
22	M	CA	96	9/8/06	3/8/07	AB gait *	(1) 6/14/07 weakness; (2) 8/15/07 ABP pain; (3) 9/2/07 NOSE bleed; (4) 9/15/07 nose bleed	(1) $1542 (2) $715 (3) $765 (4) $1039
						colon diverticulosis		
						left heart failure (CHF)		
						muscle weakness		
26	F	CA	92	3/15/08	4/23/08	traum up leg FX AFTC	6/24/08 ams (altered mental status)	$1696
						rheumatoid arthriti		
						osteoporosis NOS		
						HTN NOS (CVD)		
28	F	CA	84	4/16/08	6/13/08	malaise & fatigue NE	7/15/08 abd pain dementia, incontinence	$4368
						muscle weakness		
						HTN NOS (CVD)		
						asthma NOS		
						AOTH persist ment DIS		
14	M	CA	71	2/15/07	2/22/07	DM2/NOS W circ DIS U	9/12/07 ADM: GI bleed (medication SIDE effect)	$29,380
						angiopathy IN DCE		
						HTN NOS (CVD)		
						lower limb ulcer NOS		
27	M	CA	63	4/5/08	6/15/08	OCB W exacerbation	(1) 6/21/08 dehydration (2) 7/13/08 RESP/failure, COPD, Chf	(1) $16,430 (2) $22,283
						DM1 uncomp UNC		
						atten to tracheostom		
						old myocardial infar (CVD)		
						COR AS-graft type NO		
132	F	CA	86	2/28/08	4/22/08	AB gait *	(1) 8/31/08 tia (transient ischemic attack) (2) 9/4/08 (3) 9/26/08 knee pain	(1) $10,761 (2) $10,635 (3) $988n
						multiple contusion		
						HTN NOS (CVD)		
						atrial fibrilliation		
						left heart failure (CHF)		
10	F	CA	69	9/25/07	11/8/07	left heart failure (CHF)	1/29/08 pneumonia, chf, copd	$1063
						muscle weakness		
						DM2/NOS uncomp NSU		
						HTN NOS (CVD)		
						HX mental disorder N		
34	M	CA	75	4/21/08	6/6/08	chronic kidney DIS N	(1) 7/25 blood in urine—signed out AMA1 (2) 7/28/08 anemia, CRD, hyponatremia, DM	(1) $0 (2) $5362
						COR AS-graft typre NO		
						HTN NOS (CVD)		
						DM2/NOS uncomp NSU		
						hyperlipidemia NEC		
130	F	AA	68	1/30/08	3/6/08	joint REPL aftercare	(1) 3/16/08 blood sugar problem (2) 4/7/08 dizzness	(1) $178 (2) $2911
						AB gait *		
						DM2/NOS uncomp NSU		
						malignant HTN (CVD)		
						pure hypercholesterol		
23	F	CA	78	3/18/07	6/15/07	DM2/NOS W comp NOS N	12/04/07 DM, weakness, htn	$5111
						AB gait *		
70	M	CA	62	7/8/07	9/25/07	late EFF CVD-cogniti	12/15/07 diabetic ketoacidosis, mi, cri	$23,999
						gastrostomy status		
						DM2/NOS uncomp UNC		
						DM2/NOS w neur manif		
						neuropathy		
**Sub Total:**								**$156,248**
**Average Cost of Care (per person):**								**$11,161**

* abnormality of gait.

**Table 4:** Gender, Race, Age, HHS Enrollment Dates and HHS Diagnoses of Comparison Patients with No Emergent Care (*N* = 16/36, 44.4%).

Patient	Gender	Race	Age	Enrolled in HHS	HHS Diagnosis
10806	F	CA	83	10/29/06	AB gait *
					atrial fibrilliation (CVD)
10980	F	CA	87	12/13/06	mitral valve insufficiency
					muscle weakness, CAD (CVD), AB gait *
13484	F	CA	87	6/18/08	AFIB (CVD), muscle weakness, URI, osteoporosis, AB gait *
12491	F	CA	86	6/22/08	lack of coordination, AFIB, osteoporosis (CVD)
13884	F	HS	73	8/30/08	HTN (CVD), muscle weakness, pneumonia, GERD, hyperipidemia
12437	F	CA	91	11/6/08	AB gait *, HTN (CVD)
					joint replacement aftercare
11518	M	CA	80	4/14/07	coronary atherosclerosis, aftercare circulatory surgery (CVD)
11861	F	CA	83	7/1/07	DM2 W/O comp, muscle weakness, generalized pain, osteoarthritis, AB gait *
11714	M	CA	62	5/27/07	AB gait *, DM2 W/circulatory, neuroritis
13056	F	CA	72	3/17/08	pain in limb, gait alteration, oseoarorsis, DM2
9911	F	CA	91	3/30/07	cerebral thrombosis, muscle weakness, DM2, HTN (CVD), EDEMA
11622	F	CA	89	7/2/07	AB gait, DM2
13425	F	CA	68	6/6/08	DM2, HTN (CVD), DJC, gait alteration
12425	M	CA	71	10/27/08	DM2, CAD, muscle weakness, COPD, HX of CABG (CVD)
3275	F	CA	76	1/19/08	CHF, DM2, Alzheimer’s

* abnormality of gait.

**Table 5:** Gender, Race, Age, HHS Enrollment Dates, HHS Diagnosis, Causes and Costs of Emergent Care of Comparison Patients (*N* = 22/36, 61.1%).

Patient	Gender	Race	Age	Enrolled in HHS	HHS Diagnosis	ED/Hospital Admission	Cost of Care
10608	F	CA	91	9/14/06	IDDM, obesity, CHF, HTN	(1) 10/5/07 pneumonia (2) 10/20/07 FLU like symptoms	(1) $46,250 (2) $917
11067	F	CA	73	7/26/07	DM2/neuroab gait *, pain in spine	(1) 8/1/07 nausea/vomiting (2) 9/2/07 rofound weakness	(1) $1433 (2) $5214
12723	F	CA	62	1/5/08	syncope/collapse, dressing changes, AB gait *, HX falls, DM	3/14/08 seizure	$13,233
11574			84	1/8/08	DM	(1) 3/16/08 fall (2) 4/30/08 leg pain (3) 5/1/08 leg pain (4) 5/6 OPO CVA/TIA (5) 5/18, (6) 6/9/08 TIA (7) 7/20/08 constipat	(1) $1892 (2) $2370 (3) $1523 (4) $9534 (5) $2983 (6) 23,837 (7) $548
12863	M	CA	83	2/5/08	aftercare circulatory, CAD, AFIB, HTN, AB gait *, hyperlipidemia (CVD)	2/7/08 groin pain/swelling	$639
13745	F	CA	63	8/6/08	COPD, HTN (CVD), reflux, obesity	9/8/08 RIB/hand pain	$3618
10508			87	8/24/06	DM	(1) 1/4/07 fall (2) 1/22/08 CVA deceased	(1) $11,600 (2) $29,661
11130	M	CA	85	1/18/07	CAD, CHF, dementia	9/07 syncope *	$363
11776	M	CA	73	6/13/07	irregular heart rate, LOW B/P (CVD)	6/29/07 diabetes, wound HTN	$1780
11255	F	CA	82	2/20/07	AFIB (CVD), CABG, CHF	7/22/07 EMS low blood sugar	$367
12179			73	2/13/08	DM	8/29/08 TIA	$31,327
10344	M	CA	80	7/14/06	CHF, IDDM/renal manif, CKD STGE 4, sinoatrial node dys (CVD)	(1) 11/13/06 hyperkalemia, CKD, DM (2) 11/17/06 EMS RESP/distress/deceased	(1) $3835 (2) $722
13175			61	4/14/08	CVD	(1) 7/15/08 hand injury (2) 9/20/08 AFIB, pnemonia, CHF	(1) $130 (2) $65,608
9298	M	CA	87	11/30/6	DM2, HTN (CVD)	(1) 9/20/07 falls (2) 9/29/07 dsypnea *, R/O PE (3) 9/5/07 CHF, AFIB,COPD	(1) $1970 (2) $6047 (3) $47,392
11267	F	CA	72	2/21/08	CHF, HTN, surgery circulatory, DM2, renal failure	(1) 6/18/08 CHF, HTN (2) 7/12/08 backpain (3) 8/19/08 breast pain	(1) $12,493 (2) $292 (3) $724
11229	F	AA	77	2/13/07	DM2, CHF, HTN (CVD), AB gait * hyperlipidemia	(1) 5/13/07 elevated blood sugar (2) 6/18/08 elevated blood sugar (3) 9/27/08 respiratory failure	(1) $755 (2) $28,379 (3) $40,737
12154			73	9/6/07	CHF	4/18/08 cellulitis *, pnemonia, COPD, CVD	$19,721
5155	F	CA	72	3/23/07	CAD (CVD)	7/25/07 OPO chest pain	$13,846
10549			73	8/30/06	DM	12/6/06 cellulitis *, osteomyelitis, DM, diabetic foot wound/amputation	$23,347
13077	M	CA	64	4/28/08	emphesma, DM2, CHF, AB GAIT *, AFIB (CVD)	6/26/08 COPD, pneumonia	$18,794
**Sub Total:**							**$493,064**
**Average Cost of Care (per person):**							**$23, 479.24**

* abnormality of gait.

**Table 6:** Emergent Care for Pneumonia, Flu and Falls—Comparison Patients and HC Clients.

Patient	Enrolled in HHS	HHS Diagnoses	ED/Hospital Admission Date/Cause	Other Care
12154	9/6/07	CHF	4/18/08 cellulitis pneumonia, COPD, CVD	Yes
13175	4/14/08	CVD	9/20/08 AFIB, pneumonia, CHF	Yes
10608	9/14/06	DM, CVD, CHF	10/05/07 pneumonia	Yes
			10/20/07 FLU	
13077	4/24/08	DM, CVD, CHF	6/26/08 COPD, pneumonia	Yes
11574	1/08/08	DM	3/16/08 fall	Yes
10508	8/24/06	DM	1/04/07 fall	Yes
9298	11/30/06	DM, CHF	9/20/07 fall	Yes
9280	10/25/07	DM, CHF	9/20/07 fall	No
13343	5/19/08	DM, CVD	8/27/08 fall	No
HC Client	Enrolled in HHS Enrolled in HC	HHS Diagnoses	ED/Hospital Admission Date/Cause	
10	9/25/07	CHF, DM, CVD	1/29/08 PNEUMONIA CHF, COPD	Yes
	11/08/07			

**Table 7:** ED/Hospital Visit Rate and Average Visit Costs per Patient, Comparison versus Treatment Groups.

	Average # ED/Hospital Visits		Average Costs		Percent Persons with ED/Hospital Visit	
	90 Day	180 Day	90 Day	180 Day	90 Day	180 Day
Comparison	0.28	0.72	$1135.9	$7203.68	24%	44%
Treatment	0.13	0.29	$770.55	$2545.38	10%	19%

**Table 8:** Range of Ed/Hospital Visit Costs per Treatment and Comparison Patients.

	Comparison	Treatment
Min	$393	$1698
Q1	$1272	$3409
Median	$7112	$4865
Q3	$20,723	$20,600
Max	$65,738	$38,713

## References

[1] US Census Bureau (2016). US Census Bureau, Population Division, Annual Estimates of the Resident Population for Selected Age Groups by Sex for the United States, States, Counties, and Puerto Rico Commonwealth and Municipios.

[2] Ward B.W., Schiller J.S., Goodman R.A. (2014). Multiple Chronic Conditions among US Adults: A 2012 Update. Prev. Chronic Dis..

[3] National Institute on Aging (2016). Aging Well in the 21st Century: Strategic Directions for Research on Aging. https://www.nia.nih.gov/sites/default/files/2017-07/nia-strategic-directions-2016.pdf.

[4] Moy E., Garcia M.C., Bastian B., Lauren M., Rossen D.D., Ingram M.F., Greta M., Massetti C.C., Hong Y., Iademarco M.F. (2017). Leading Causes of Death in Nonmetropolitan and Metropolitan Areas—United States, 1999–2014. MMWR Surveill. Summ..

[5] Meit M., Knudson A., Gilbert T., Tanenbaum E., Ormson E., TenBroeck S., Bayne A., Popat S. (2014). The 2014 Update of the Rural Urban Chartbook.

[6] Roberts M.E., Doogan N.J., Kurti A.N., Rednerb R., Gaalema D.E. (2016). Rural tobacco use across the United States: How rural and urban areas differ broken down by census regions and divisions. Health Place.

[7] Patterson P.D., Moore C.G., Probst J.C. (2004). Obesity and physical inactivity in rural America. J. Rural Health.

[8] Befort C.A., Nazir N., Perri M.G. (2012). Prevalence of obesity among adults from rural and urban areas of the United States; findings from NHANES (2005–2008). J. Rural Health.

[9] Fan J.X., Wen M., Kowaleski-Jones L. (2014). Rural-urban differences in objective and subjective measures of physical activity: Findings from the National Health and Nutrition Examination Survey (NHANES) 2003–2006. Prev. Chronic Dis..

[10] Dixon M.A., Chartier K.G. (2016). Alcohol use patterns among urban and rural residents: Demographic and social influences. Alcohol Res..

[11] National Center for Health Statistics (2016). Health, United States 2015: With Special Feature on Racial and Ethnic Disparities.

[12] James W.L. (2014). All rural places are not created equal: Revisiting the rural mortality penalty in the United States. Am. J. Public Health.

[13] Anderson T.J., Saman D.M., Lipsky M.S. (2015). A cross-sectional study on health differences between rural and non-rural counties using the County Health Rankings. BMC Health Serv. Res..

[14] Caldwell J.T., Ford C.L., Wallace S.P. (2016). Intersection of living in rural versus urban area and race/ethnicity in explaining access to health care in the United States. Am. J. Public Health.

[15] Cohen J.W., Krauss N.A. (2003). Spending and service use among people with the fifteen most costly medical conditions, 1997. Health Aff..

[16] Goetzel R.Z., Hawkins K., Ozminkowski R.J., Wang S. (2003). The health and productivity cost burden of the “top 10” physical and mental health conditions affecting six large U.S. employers in 1999. J. Occup. Environ. Med..

[17] Thorpe K.E., Florence C.S., Joski P. (2004). Which medical conditions account for the rise in health care spending?. Health Aff..

[18] Roehrig C., Miller G., Lake C. (2009). National health spending by medical condition, 1996–2005. Health Aff..

[19] Trogdon J.G., Tangka F.K., Ekwueme D.U., Bryant J. (2012). State-level projections of cancer-related medical care costs: 2010 to 2020. Am. J. Manag. Care.

[20] Centers for Medicare and Medicaid Services Community—Based Care Transitions Program. https://partnershipforpatients.cms.gov/about-the-partnership/community-based-care-transitions-program/community-basedcaretransitionsprogram.html.

[21] Boccuti C., Casilllas G. (2015). Aiming for Fewer Hospital U-Turns: The Medicare Hospital Readmission Reduction Program.

[22] Ritsema T.S., Bingenheimer J.B., Scholting P. (2014). Differences in the Delivery of Health Education to Patients with Chronic Disease by Provider Type, 2005–2009. Prev. Chronic Dis..

[23] Earp J.L., Flax V. (1999). What lay health advisors do: An evaluation of advisors’ activities. Cancer Pract..

[24] Dohan D., Schrag D. (2005). Using navigators to improve care of underserved patients: Current practices and approaches. Cancer.

[25] Brownstein J.N., Bone L.R., Dennison C.R., Hill M.H., Kim M.T., Levine D.V. (2005). Community health workers as interventionists in the prevention and control of heart disease and stroke. Am. J. Prev. Med..

[26] Brownstein J., Allen C. (2015). Addressing Chronic Disease through Community Health Workers: A Policy and Systems—Level Approach.

[27] Kivelä K., Elo S., Kyngäs H., Kääriäinen M. (2014). The effects of health coaching on adult patients with chronic diseases: A systematic review. Patient Educ. Couns..

[28] Wong-Rieger D., Rieger F.P. (2013). Health coaching in diabetes: Empowering patients to self-manage. Can. J. Diabetes.

[29] Centers for Medicare and Medicaid Services. https://www.cms.gov.

[30] Freeman H.P., Rodriguez R.L. (2011). History and principles of patient navigation. Cancer.

[31] Hewner S. (2014). A Population-Based Care Transition Model For Chronically III Elders. Nurs. Econ..

[32] Kaspin L.C., Gorman K.M., Miller R. (2013). Systematic review of employer-sponsored wellness strategies and their economic and health-related outcomes. Popul. Health Manag..

[33] Rosenthal E.L., Wiggins N., Ingram M., Mayfield-Johnson S., Mayfield-Johnson J.E. (2011). Community health workers then and now: An overview of national studies aimed at defining the field. J. Ambul. Care Manag..

[34] Wolever R.Q., Eisenberg D. (2011). What Is Health Coaching Anyway? Standards Needed to Enable Rigorous Research: Comment on “Evaluation of a Behavior Support Intervention for Patients with Poorly Controlled Diabetes”. Arch. Int. Med..

[35] Health Resources & Services Administration, Federal Office of Rural Health Policy (2007). Community Health Coaches for Successful Care Transitions”. https://www.ruralhealthinfo.org/community-health/project-examples/375.

[36] Dye C.J., Willoughby F., Battisto D. (2011). Advice from Rural Elders: What it Takes to Age in Place. Educ. Gerontol..

[37] Wagner E.H., Austin B.T., Davis C., Hindmarsh M., Schaefer J., Bonomi A. (2001). Improving chronic illness care: Translating evidence into action. Health Aff..

[38] Hollwitz J., Wilson C.E. (1993). Structured interviewing in volunteer selection. J. Appl. Commun. Res..

[39] Lorig K.R., Holman H.R. (2003). Self-management education: History, definition, outcomes, and mechanisms. Ann. Behav. Med..

[40] Cohen D.J., Balasubramanian B.A., Davis M., Hall J., Gunn R., Stange K.C., Green L.A., Miller W.L., Crabtree B.F., England M.J. (2015). Understanding care integration from the ground up: Five organizing constructs that shape integrated practices. J. Am. Board Fam. Med..

[41] Released 2015. IBM SPSS Statistics for Windows.

[42] Perry Undem Research & Communications (2013). The Revolving Door: A Report on US Hospital Readmissions. http://www.rwjf.org/en/library/research/2013/02/the-revolving-door-a-report-on-u-s-hospital-readmissions.html.

[43] Naylor M.D., Aiken L.H., Kurtzman E.T. (2011). The importance of transitional care in achieving health reform. Health Aff..

[44] Coleman E.A., Parry C., Chalmers S. (2006). The care transitions intervention: Results of a randomized controlled trial. Arch. Int. Med..

[45] Russell D., Mola A., Onorato N., Johnson S., Williams J., Andaya M., Flannery M. (2017). Preparing home health aides to serve as health coaches for home care patients with chronic illness: Findings and lessons learned from a mixed-method evaluation of two pilot programs. Home Health Care Manag. Pract..

